# Selective killing of HIV-1-positive macrophages and T cells by the Rev-dependent lentivirus carrying *anthrolysin O *from *Bacillus anthracis*

**DOI:** 10.1186/1742-4690-5-36

**Published:** 2008-04-25

**Authors:** Jessica Young, Zhongwei Tang, Quan Yu, Dongyang Yu, Yuntao Wu

**Affiliations:** 1 Department of Molecular and Microbiology, George Mason University, Manassas, VA 20110, USA; 2 College of Life Sciences, Shanxi Agricultural University, Taigu, Shanxi 030801, P. R. China; 3 Advanced Vision Therapies, Inc., Gaithersburg, MD 20878, USA

## Abstract

**Background:**

The ability of Human Immunodeficiency Virus (HIV) to persist in the body has proven to be a long-standing challenge to virus eradication. Current antiretroviral therapy cannot selectively destroy infected cells; it only halts active viral replication. With therapeutic cessation or interruption, viral rebound occurs, and invariably, viral loads return to pre-treatment levels. The natural reservoirs harboring replication-competent HIV-1 include CD4 T cells and macrophages. In particular, cells from the macrophage lineage resist HIV-1-mediated killing and support sustained viral production. To develop a complementary strategy to target persistently infected cells, this proof-of-concept study explores an HIV-1 Rev-dependent lentiviral vector carrying a bacterial hemolysin, *anthrolysin O *(*anlO*) from *Bacillus anthracis*, to achieve selective killing of HIV-1- infected cells.

**Results:**

We demonstrate that in the Rev-dependent lentiviral vector, *anlO *expression is exclusively dependent on Rev, a unique HIV-1 protein present only in infected cells. Intracellular expression and oligomerization of AnlO result in membrane pore formation and cytolysis. We have further overcome a technical hurdle in producing a Revdependent AnlO lentivirus, through the use of β-cyclodextrin derivatives to inhibit direct killing of producer cells by AnlO. Using HIV-1-infected macrophages and T cells as a model, we demonstrate that this Rev-dependent AnlO lentivirus diminishes HIV-1- positive cells.

**Conclusion:**

The Rev-dependent lentiviral vector has demonstrated its specificity in targeting persistently infected cells. The choice of *anlO *as the first suicidal gene tested in this vector is based on its cytolytic activity in macrophages and T cells. We conclude that Rev-regulated expression of suicidal genes in HIV-1-positive cells is possible, although future *in vivo *delivery of this system needs to address numerous safety issues.

## Background

The success of highly active antiretroviral therapy (HAART), marked by the drastic reduction of plasma viremia and restoration of certain immune functions [1-3], led initially to speculation of disease eradication in 2 to 3 years [4,5]. This original optimism was soon dampened by the realization that persistence of viral reservoirs would make it extremely difficult, if not impossible, to eradicate HIV-1 [6-11]. Further identification and characterization of these reservoirs have highlighted the limitations of HAART. It has become evident that with drug cessation, viral loads return to pre-HAART levels [12,13]. With no alternative approaches in use to specifically target cells harboring the virus, it would take an estimated 60 years for some of these reservoirs to naturally decay [14].

The primary reservoirs of HIV-1 include resting CD4 T cells and cells of the macrophage lineage. Both are the natural targets of HIV-1. It has been shown that *in vitro *stimulation of resting CD4 T cells from patients receiving HAART can recover replication-competent virus [6-8]. In these cells, HIV-1 exists primarily as a postintegrated latent form with no detectable viral gene expression in the absence of stimulation. Nevertheless, low-level ongoing viral replication may occur in the body even with concurrent HAART [15-17]. In particular, with the cessation of therapy, rebounding plasma viruses do not entirely reflect the genetic pool of the viruses from resting CD4 T cells [18,19], suggesting the existence of other reservoirs such as cells from the macrophage lineage [15-17]. In contrast to the latent reservoir of resting T cells, macrophages are metabolically active and support sustained, ongoing viral replication [20]. Macrophages have minimal cytopathology in response to HIV infection and can remain viable for viral production for extended periods of time [21,22]. In addition, antiretroviral drugs are poorly efficacious against chronically infected macrophages [22-24]. These features suggest that macrophages are a major viral reservoir in the body [10,11,17,22,25] and an attractive target for testing alternative therapies aimed at eradicating HIV reservoirs.

Clinical and experimental attempts to diminish HIV reservoirs have taken many forms. For example, infected patients have been treated with HAART plus cytokines such as IL-2 and INF-γ [26-29], or the chemical compound valproic acid [30,31], in hopes of purging the latent T cell reservoir via activation-driven killing, either by the virus itself or by immune effector mechanisms. Others have opted for more aggressive approaches to counter the HIV-infected cells, such as targeting the cells with hybrid CD4-toxins that can bind to the viral envelope [32]. More recently, an HIV LTR-based lentiviral vector expressing herpes simplex virus thymidine kinase (TK) has been used to inhibit HIV replication in a latently infected T cell line [33]. Nevertheless, a major limitation in many of these approaches is the lack of high specificity required to target only HIV-infected cells.

In the pursuit of eliminating viral reservoirs, as a proof of concept, this study offers a solution utilizing an engineered Rev-dependent lentivirus to achieve high specificity [34]. This lentiviral vector utilizes the Rev responsive element (RRE), which renders gene expression dependent on Rev, a viral early protein interacting specifically with RRE to mediate mRNA nuclear export and translation [35]. Using the green fluorescent protein (GFP) as a reporter gene, we have demonstrated that this Rev-dependent vector, when assembled into a viral particle and delivered into target cells, is fully dependent on HIV with no detectable background expression in uninfected cells [34,36]. However, this attractive lentivirus was unable to deliver highly effective cytotoxic or cytolytic genes into HIV-1-infected cells since these genes can directly kill the producer cells, thereby preventing lentiviral particle production *in vitro*. In this study, we have used β-cyclodextrin derivatives to overcome this technical hurdle and successfully generated a Rev-dependent lentivirus carrying a bacterial cytolytic gene, Anthrolysin O (*anlO*), and used it to target HIV-1-infected macrophages.

AnlO is a thiol-activated hemolysin from the bacterium *Bacillus anthracis*. The thiol-activated hemolysins are a family of cytolysins expressed by 15 diverse bacterial species. Features common to these hemolysins include inhibition by free cholesterol and the presence of a unique cysteine residue that renders the hemolysins susceptible to reverse inactivation by oxidation. The mechanism of hemolysin action is thought to involve an oligomerization of 20 to 80 monomers into ring and arch-like structures that aggregate within the cell membrane and form large pores [37,38]. The most compelling evidence for a direct role of thiol-activated lysins in cell killing came from studies on Listeriolysin O (LLO) in *Listeria monocytogenes *infection. It has been shown that a PEST-like motif at the N-terminus of LLO is responsible for its unique ability to lyse phagosomal but not cytoplasmic membrane [39]. Upon their release from the phagosome, PEST-containing lysins are rapidly degraded by the cellular protein degradation pathway, preventing the lysins from attacking the host cell membrane and allowing the microbe to establish a productive intracellular infection. In contrast, mutants that lack the PEST-like sequence enter the host cytosol but subsequently permeabilize and kill the host cell [39]. The LLO analog, AnlO, expressed by *B. anthracis *is highly homologous to LLO (37% identity). The ability to escape the phagosome of macrophages is also a characteristic feature of *B. anthracis*. However, in contrast to the non-cytolytic nature of *L. monocytogenes *infection, *B. anthracis *infection results in the death of infected macrophages. Consistently, the AnlO sequence contains no PEST homology, and AnlO kills macrophages likely by direct lysis of the cell membrane.

The unique features of LLO have been used for the delivery of gelonin toxin into tumor cells for therapeutic purposes [40]. In *in vitro *experiments, co-encapsulated LLO enabled the release of liposomal gelonin into cell cytoplasm, resulting in rapid cell killing by gelonin. Conceivably, in this Rev-dependent lentiviral system, AnlO would be superior to LLO, because cytosolic AnlO can cause cell death even in the absence of gelonin. This study is the first to test the feasibility of using AnlO as a therapeutic tool to target HIV-1-infected macrophages and T cells.

## Results

### Construction of the Rev-dependent lentiviral vector carrying *anthrolysin O *from *Bacillus anthracis*

**Figure 1:**
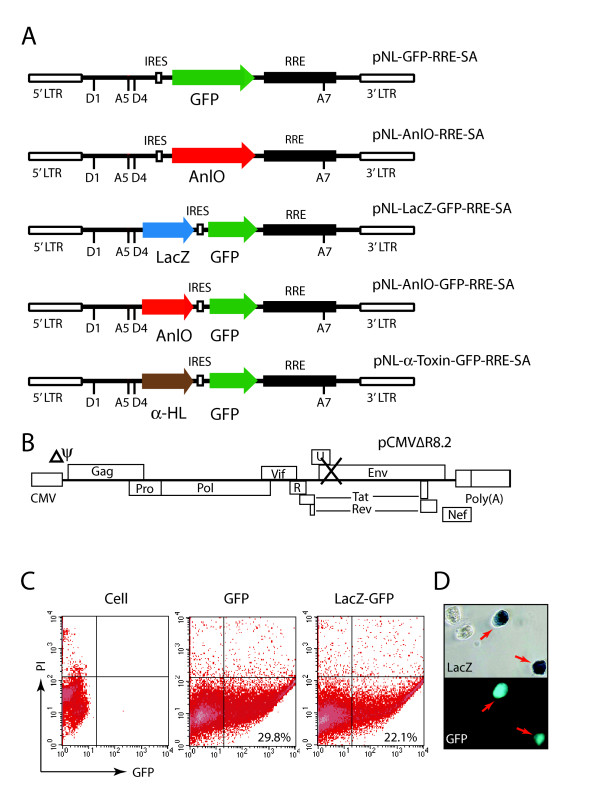
**Construction of the Rev-dependent lentiviral vectors**. (A) Schematic representation of the Rev-dependent lentiviral constructs. Shown are pNL-GFP-RRE-SA and its derivatives. The HIV-1 5' LTR, packaging signal (ψ), splice donors (D1, D4) and acceptors (A5, A7), IRES, RRE, and 3' LTR are indicated. The Rev-dependent constructs would transcribe both spliced and unspliced transcripts as HIV-1 does. Only the unspliced or partially spliced transcripts that contain reporters or toxins are Rev-dependent for expression. α-HL is the α-hemolysin of *Staphylococcus aureus*. (B) Schematic representation of the HIV-1 helper construct, pCMVΔR8.2, in which both the viral package signal (Δψ)and the envelope gene (Env) were deleted. (C) Simultaneous expression of two genes via IRES in the Rev-dependent construct. Both the *E. coli lacZ *and the GFP genes were cloned into the vector, pNL-lacZ-GFP-RRE-SA, which was cotransfected with pCMVΔ8.2 into HEK293T cells. For comparison, pNL-GFP-RRE-SA was similarly cotransfected. Shown is GFP expression measured by flow cytometry at 48 hours post cotransfection with either pNL-GFP-RRE-SA (middle panel, GFP) or pNL-lacZ-GFP-RRE-SA (right panel, LacZ-GFP). (D) Cells from pNL-LacZ-GFP-RRE-SA cotransfection were also stained for LacZ and visualized under the microscope for LacZ and GFP. One hundred cells were counted, and among them, 20 expressed both LacZ and GFP; 4 expressed only LacZ; and 76 expressed neither LacZ nor GFP.

The Rev-dependent lentiviral vector is structurally based upon the HIV-1 genome and has been described previously [34,36] (Fig. 1A). As shown in Fig. 1A, we placed the GFP or the *anlO *gene under the control of Rev by introducing multiple splicing sites and an RRE. This arrangement would regulate these genes as late genes and render their expression highly specific to Rev. The lentiviral vector also contains an internal ribosome entry site (IRES) that allows the expression of two genes simultaneously. To demonstrate the operation of the IRES, we inserted both the *E. coli lacZ *and the GFP gene into a single vector. This construct, pNL-LacZ-GFP-RRE-SA (Fig. 1A), was then cotransfected with an HIV-1 helper plasmid, pCMVΔ8.2 (Fig. 1B) [41], which provides both Tat and Rev to mediate the expression of LacZ and GFP in the same cell. Indeed, in the cotransfected HEK293T cells, co-expression of LacZ and GFP was detected with the help of pCMVΔ8.2 (Fig. 1C and 1D). The IRES feature was also implemented in this study to clone *anlO *in front of GFP (pNL-AnlO-GFP-RRE-SA). This would permit us to monitor AnlO-mediated lysis of HIV-positive cells by directly measuring the reduction of GFP expression; because only 30 molecules of AnlO are required to trigger cytolysis [42], GFP would not be able to accumulate in cells that also express AnlO.

**Figure 2:**
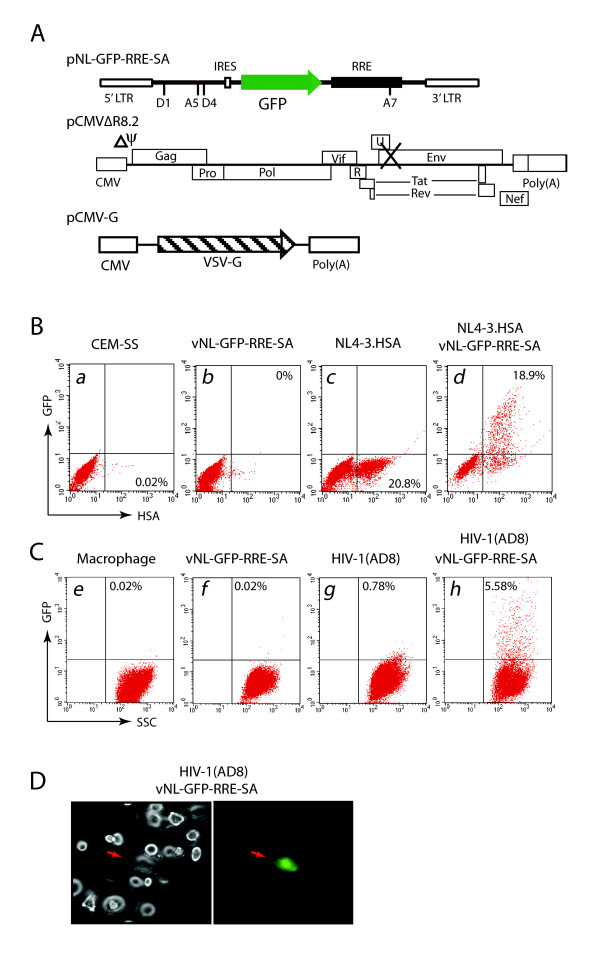
**Specificity of the Rev-dependent lentiviral vector in HIV-1- positive T cells and macrophages**. (A) Schematic representation of the three constructs used to generate the Rev-dependent GFP lentivirus. HEK293T cells were cotransfected with pNL-GFP-RRE-SA, pCMVΔ8.2, and the VSV-G construct. Viruses were harvested, concentrated, and used to infect a human T cell line, CEM-SS, as well as primary human macrophages. (B) Specificity of the Rev-dependent lentiviral vector in HIV-1-positive T cells. CEM-SS cells were not infected (*a*) or infected with NL4-3.HSA.R+E-(VSV-G) (*d*, 500 ng p24 per million cells), a VSV-G pseudotyped HIV-1 strain with the murine heat-stable antigen CD24 (HSA) gene inserted into the *nef *region that allows HIV-1-positive cells to be monitored by surface staining of HSA. At 24 hours, cells were superinfected with lentivirus vNL-GFP-RRE-SA (*d*, m.o.i. 10). For comparison, cells were also singly infected with either vNL-GFP- RRE-SA (*b*) or NL4-3.HSA.R+E-(VSV-G) (*c*). At 72 hours, cells were harvested, stained with a PE-labeled rat monoclonal antibody against mouse CD24 (HSA), and then analyzed on a flow cytometer for both HSA and GFP expression. Isotype staining was not shown. (C) Specificity of the Rev-dependent lentiviral vector in HIV-1-positive macrophages. Human macrophages were derived from peripheral monocytes by culturing in 10 ng/ml M-CSF for two weeks. Cells were not infected (*e*) or infected with HIV- 1(AD8) (*h*, 380 ng p24 per million). At 24 hours, cells were superinfected with lentivirus vNL-GFP-RRE-SA (*h*, m.o.i. 10). For comparison, cells were also singly infected with either vNL-GFP-RRE-SA (*f*) or HIV-1(AD8) (*g*). At 72 hours, cells were harvested and analyzed on a flow cytometer for GFP expression. (D) Fluorescent microscopy of GFP expression in macrophages infected with HIV-1 and the Rev-dependent GFP lentiviral vector. Cells in (C, h) were also examined with fluorescent microscope. The left and right panels show the bright and green fluorescent fields of the same cells. Red arrows indicate an HIV-1-infected cell expressing the GFP protein.

Previously, using GFP as a reporter, we demonstrated that the Rev-dependent lentivirus can mark 80–90% of HIV-1-infected cells [34]. To further test the specificity of the Rev-dependent vector to express genes both in primary human macrophages and in T cells, we produced and tested a Rev-dependent GFP lentivirus, vNL-GFP-RRE-SA. Viral particles were generated by cotransfection of HEK293T cells with pNL-GFP-RRE-SA [34], pCMVΔ8.2, and a construct expressing the VSV-G envelope (Fig. 2A). Concentrated particles were then used to infect a human T cell line, CEM-SS, or human monocyte-derived macrophages, either directly or following infection with HIV-1. For HIV-1 infection, CEM-SS were infected with NL4-3.HSA.R+E- (Vpr^+^, Env^-^), a VSV-G pseudotyped HIV strain with the murine heat stable antigen CD24 (HSA) gene inserted into the *nef *region to facilitate surface HSA staining of HIV-1-positive cells [43]. Macrophages were similarly infected with an M-tropic virus, HIV-1(AD8) [44]. As shown in Fig. 2B to 2D, while vNL-GFP-RRE-SA effectively targeted HIV-1-infected cells, this lentiviral vector did not generate any GFP-positive cell without HIV-1 coinfection even with a vector multiplicity of infection as high as 10.

### Extracellular and intracellular cytolytic activity of AnlO

**Figure 3:**
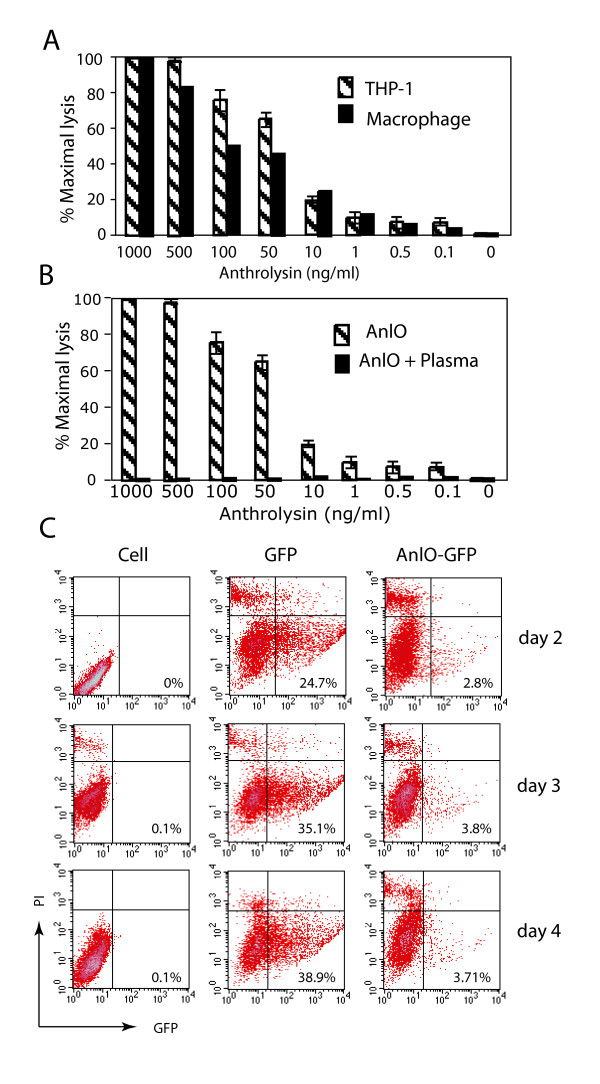
**Extracellular and intracellular cytolytic activity of AnlO**. (A) Extracellular cytolytic activity of AnlO. THP-1 cells or primary human macrophages were treated with different concentrations of purified AnlO in serum-free medium for one hour at 37°C. Cytolytic activity of AnlO was assayed by measuring the relative activity of lactate dehydrogenase (LDH) released into the culture following cell lysis. (B) Inhibition of AnlO activity by human plasma. AnlO was serially diluted and incubated with human plasma (heat inactivated) for 20 minutes on ice and then added to THP-1 cells in serumfree medium for one hour at 37°C. Cytolytic activity of AnlO was assayed by measuring the relative activity of lactate dehydrogenase (LDH) released into the culture. In the controls, AnlO was directly added into THP-1 cells without being neutralized by plasma. (C) Intracellular cytolytic activity of AnlO. pNL-GFP-RRE-SA (middle panels, GFP) or pNL-AnlO-GFP-RRE-SA (right panels, AnlO-GFP) was co-transfected with pCMVΔ8.2 into HEK293T cells. At day 2 to 4 after cotransfection, cells were analyzed by flow cytometry for GFP expression. PI is propidium iodide. Mock transfected cells (left panels, Cell) were used as controls for the GFP and PI positive population.

To demonstrate that AnlO is capable of killing HIV-1 target cells such as macrophages, we used a monocyte-like cell line, THP-1, as well as primary human monocyte-derived macrophages. Cells were treated with increasing concentrations of purified AnlO for one hour and cytolysis was measured by lactate dehydrogenase (LDH) release into the cell culture. As shown in Fig. 3A, we found that in both cell types the extent of cytolysis correlates with the toxin concentration. Others have also demonstrated that highly purified AnlO is extremely cytolytic against human erythrocytes, with only 30 molecules required for cell lysis [42]. This highly cytolytic activity of AnlO would be advantageous in targeting HIV-1-positive cells, since minimal gene expression is required. Another benefit of using AnlO is that it is not an enzyme. Upon its oligomerization, AnlO inserts itself into the cell membrane and remains membrane-bound. Following cell lysis, the AnlO oligomer is not expected to re-activate, minimizing possible lysing of healthy bystander cells. Nevertheless, despite the high specificity provided by the Rev-dependent lentivirus, possible residual AnlO present in cell lysates might expose healthy cells to the toxin. To test whether the AnlO released from cells could induce damage to healthy bystanders, we incubated the cell lysates (lysed with 1 μg/ml AnlO) with healthy macrophages. We did not observe further cell lysis, thereby indicating minimal cytolytic effects on uninfected cells (data not shown). It has also been shown that AnlO is highly sensitive to inhibition by cholesterol [42] which is abundantly present in human blood plasma. We then examined whether human plasma is sufficient to neutralize AnlO. Incremental concentrations of AnlO were pre-incubated with human plasma for 20 minutes on ice before being added to THP-1 cells. We observed complete inhibition of AnlO cytolytic activity at all AnlO concentrations tested (Fig. 3B), demonstrating the ability of blood plasma to neutralize AnlO. The susceptibility of AnlO to human plasma would add a second layer of protection from possible unforeseeable bystander killing.

To further test cytolysis by intracellular delivery of AnlO, via the Rev-dependent lentiviral vector, cells were cotransfected with the HIV-1 helper construct, pCMVΔR8.2, and either pNL-AnlO-GFP-RRE-SA or a control plasmid, pNL-GFP-RRESA (Fig. 3C). The degree of cell lysis from *anlO *expression was measured by comparing GFP expression in the two parallel cotransfection procedures. As we mentioned above, the reduction in GFP positive population was used as an indicator for AnlO-mediated cytolysis. As shown in Fig. 3C, at day 2, cells cotransfected with pNL-AnlO-GFP-RRE-SA generated a much lower percentage (AnlO-GFP, 2.8%) of GFP positive cells than the control cells that were cotransfected with pNL-GFP-RRE-SA (GFP, 24.7%). Additionally, the GFP intensity in cells cotransfected with AnlO-GFP was also lower (mean GFP intensity: 352.99 versus 1397.02). At days 3 and 4, we observed little increase in the number of GFP-positive cells cotransfected with AnlO-GFP (from 2.8% to 3.8%). In contrast, we detected a significant increase of GFP positive cells in the control cotransfection (from 24.7% to 38.9%) (Fig. 3C). Similar results were obtained from three independent co-transfection experiments (data not shown). The diminished GFP expression in pNL-AnlO-GRP-RRE-SA cotransfection did not result from differences in cotransfection efficiency or from reduced gene expression mediated by IRES, as demonstrated in three ways. Firstly, measurements of the amount of plasmid DNA extracted from cells immediately following cotransfection revealed no significant difference from that of the control cotransfection (data not shown). Secondly, the addition of β-cyclodextrin derivatives, which block cell membrane pores and inhibit AnlO mediated cytolysis [45,46], led to a significant increase in the GFP positive cells in pNL-AnlO-GFP-RRE-SA cotransfection (see text below), whereas the same compound had little effect on cells cotransfected with the control plasmid (pNL-GFP-RRE-SA) (data not shown). Thirdly, with the cloning of multiple genes and toxins into the Rev-dependent vector, we consistently observed that diminished expression of GFP from IRES always closely correlated with the cytotoxicity of co-expressed genes. For example, when *lacZ *was co-expressed with GFP via IRES, we observed an approximately equal number of GFP-positive cells regardless of the presence of the *lacZ *gene. In contrast, when a highly cytotoxic gene, diphtheria toxin (DT) (one molecule would kill a cell) [47], was placed in front of GFP, not a single GFP-positive cell was detected (data not shown). Additionally, when the same DT constructs were cotransfected into a diphtheria toxin-resistant cell line, an equal number of GPF positive cells were observed regardless of the presence of the DT gene (data not shown). Based on these observations, we conclude that similar to the DT cotransfection, the diminished expression of GFP in pNL-AnlO-GFP-RRE-SA cotransfection correlates with AnlO-mediated cytolysis.

### Production of lentiviral particles carrying the *anlO *gene

**Figure 4:**
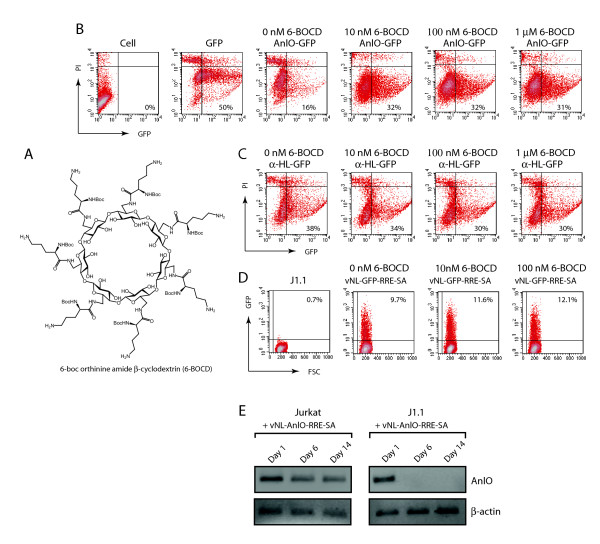
**Inhibition of AnlO-mediated cytolysis of producer cells by β-cyclodextrin derivatives**. (A) Structure of the membrane pore blocker 6-boc orthinine amide-β-cyclodextrin (6-BOCD). (B) Inhibition of AnlO-mediated cytolysis by 6-BOCD. HEK293T cells were cotransfected with pCMVΔR8.2 and either pNL-GFP-RRE-SA (labeled as GFP) or pNL-AnlO-GFP-RRE-SA (labeled as AnlO-GFP) in the absence or presence of various doses of 6-BOCD. Increases in viable GFP cells were measured by flow cytometry. (C) Lack of effect of 6-BOCD on α-hemolysin. Cells were similarly cotransfected with pCMVΔR8.2 and pNL-α-HL-GFP-RRE-SA (labeled as α-HL-GFP) in the absence or presence of various doses of 6-BOCD. (D) No inhibition of 6-BOCD on viral infectivity. Lentiviral particles, vNL-GFP-RRE-SA, were generated by cotransfection of HEK293T cells with pNL-GFP-RRE-SA, pCMVΔR8.2, and pCMV-VSV-G in the absence or presence of different concentrations of 6-BOCD. The resulting viral particles were used to infect an HIV-1-positive cell, J1.1 (using an equal p24 level of viruses, 150 ng p24 per million cells). The percentage of GFP positive J1.1 cells was used as an indicator for viral infectivity. (E) The Rev-dependent AnlO lentiviral vector is suicidal in HIV-1-positive cells. The HIV-1-positive cell, J1.1, was infected with vNL-AnlO-RRE-SA (300 ng p24 per million cells). As a control, HIV-1-negative Jurkat cells were identically infected. Following infection, cells were harvested at different times and total cellular DNA was extracted and PCR amplified with primers for the *anlO *gene (AnlO). As a control, the DNA was also amplified with primers for the cellular β-actin pseudogene (β-actin) to ensure that the same number of cells was used.

The demonstration of the intracellular cytolytic activity of AnlO suggested a possible application of AnlO in killing HIV-1-positive cells. However, its cytolytic activity also presented an immediate problem in lentiviral production. Cotransfection with pCMVΔR8.2 is required to produce lentiviral particles, but the expression of Rev in turn allows the expression of *anlO *from the lentiviral construct. This would result in the lysis of HEK293T producer cells, diminishing viral production. To solve this problem, we took advantage of a recent report that β-cyclodextrin derivatives can partially block ion conductance through pores formed by hemolysins [45,46]. Thus, β-cyclodextrin derivatives were tested for their ability to block the plasma membrane pores induced by AnlO. We tested three β-cyclodextrin derivatives: 6-thioethylamino-β-cyclodextrin hydrochloride, 6-thiohexylamino-β-cyclodextrin hydrochloride, and 6-boc orthinine amide-β-cyclodextrin (6-BOCD) (Fig. 4A). Cells were cotransfected with pCMVΔR8.2 and pNL-AnlO-GFP-RRE-SA in the presence of various concentrations of β-cyclodextrin derivatives (data not shown). We found that 6-BOCD had the best effects on inhibition of cytolysis by AnlO, resulting in a doubling of the GFP-positive cell population (Fig. 4B).

We also tested another hemolysin, the α-hemolysin (α-HL) of *Staphylococcus aureus*, in the Rev-dependent lentiviral vector (Fig. 1A). Interestingly, although both AnlO and α-HL can form transmembrane pores, α-HL was less effective in mediating intracellular killing in comparison with AnlO (Fig. 4C). The reason is not clear, but could result from the lack an intracellular receptor for the oligomerization of α-HL which is normally delivered extracellularly [48,49]. Consistent with a lack of cell lysis by intracellular α-hemolysin, similar 6-BOCD treatment of cells cotransfected with the α-hemolysin construct (pNL-α-HL-GRP-RRE-SA) did not increase the number of GFP positive cells (Fig. 4C).

The impact of 6-BOCD on the infectivity of the resulting lentivirus was also tested (Fig. 4D). Infectious vNL-GFP-RRE-SA was generated by cotransfection of pNL-GFP-RRE-SA, pCMVΔR8.2, and a construct expressing the VSV-G envelope protein in the presence or absence of different concentrations of 6-BOCD. The resulting lentiviruses were used to infect the HIV-1-positive J1.1 cell line [50], using an equal level of p24. GFP expression in J1.1 cells was used to measure viral infectivity. The vNL-GFP-RRE-SA virus produced in the absence of 6-BOCD generated 9.69% GFP-positive cells, whereas the same virus generated in the presence of 6-BOCD at various doses (from 10 to 100 nM) showed no difference in infectivity (Fig. 4D). Therefore, a combined method of treating the producer cells with 6-BOCD and concentrating the virus through anion exchange columns and size-exclusion columns was used to produce high titer virus despite the cytotoxicity of AnlO. We were able to produce liters of the AnlO lentivirus (vNL-AnlO-RRE-SA, VSV-G pseudotyped) and concentrate them to several milliliters for the infection of HIV-1-positive cells.

To further confirm that vNL-AnlO-RRE-SA was indeed a suicidal vector in HIV-1-positive cells, we used this lentivirus to infect the HIV-1-positive J1.1 cells, and then followed the persistence of this vector in J1.1 cells. As a control, we also used vNL-AnlO-RRE-SA to infect the HIV-1-negative parental Jurkat cells. Following infection, cells were harvested at different times, and then total cellular DNA was extracted, and PCR-amplified for the detection of the AnlO lentiviral vector in these cells. As shown in Fig. 4E, while vNL-AnlO-RRE-SA persisted for as long as two weeks in the HIV-1-negative Jurkat cells, it diminished within 6 days in the infection of the HIV-1-positive J1.1 cells. These data suggest that cytolysis mediated by AnlO in HIV-1-positive cells likely led to the self-destruction of the AnlO vector. Additionally, the results that vNL-AnlO-RRE-SA was maintained in HIV-1-negative cells for weeks (Fig. 4E) or even months (data not shown) further demonstrated that possible HIV-1-independent expression of AnlO was minimal in uninfected cells.

### Specific killing of HIV-1-infected macrophages by the Rev-dependent lentiviral vector carrying *anlO*

**Figure 5:**
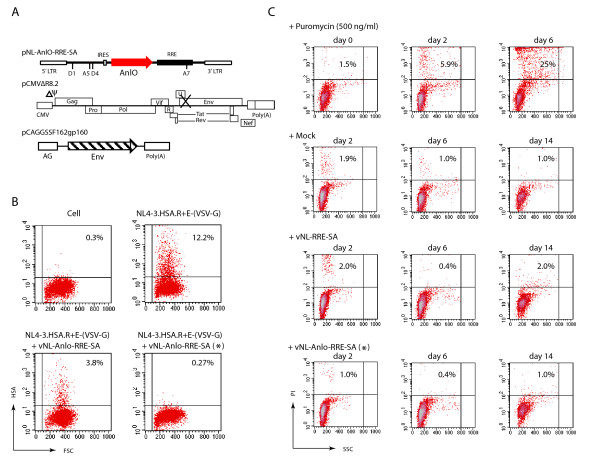
**Specific targeting of HIV-1-infected macrophages by the lentiviral vector carrying *anlO***. (A) Schematic representation of the three constructs used to generate the Rev-dependent AnlO lentivirus. HEK293T cells were cotransfected with pNL-AnlO-GFP-RRE-SA, pCMVΔ8.2, and the M-tropic envelope construct, pCAGGSSF162gp160, in the presence of 6-BOCD. Viruses were harvested, concentrated, and used to infect human macrophages. (B) Specific killing of HIV-1-positive macrophages by the Rev-dependent AnlO lentiviral vector. Human macrophages were derived from peripheral monocytes. Cells were not infected (Cell) or infected with NL4-3.HSA.R+E-(VSV-G) (m.o.i. 0.1). Following HIV infection, at 24 hours, HIV-1-infected cells were super-infected with the lentivirus vNL-AnlO-RRE-SA (approximate m.o.i. 0.5 – 1) or with the same lentivirus using a 10-fold higher dosage (*). HIV-1-infected cells were stained with a PE-labeled rat monoclonal antibody against mouse CD24 (HSA) and analyzed by flow cytometry at 10 days post infection with HIV-1. (C) Undetectable cytolytic activity of the Rev-dependent AnlO lentiviral vector in un-infected macrophages. To determine whether vNL-AnlO-RRE-SA can non-specifically kill un-infected macrophages, cells were similarly infected with highly concentrated virus (m.o.i. 5 – 10). Following infection for two weeks, cells were harvested at different times and analyzed by propidium iodide (PI) staining and flow cytometry for cytolysis. As controls, cells were also mock infected with medium, or the same dose of an empty vector virus, vNL-RRE-SA. Cells were also treated with puromycin (500 ng/ml) to induce non-specific cytolysis for the validation of PI staining and flow cytometry analysis.

To determine whether the Rev-dependent lentivirus carrying *anlO *is effective in targeting HIV-1-infected primary human macrophages, concentrated vNL-AnlO-RRE-SA was produced in the presence of 10 nM 6-BOCD by cotransfection of pNL-AnlO-RRE-SA, pCMVΔR8.2, and an M-tropic HIV envelope construct, pCAGGSSF162gp160 [51] (Fig. 5A). To demonstrate specific killing, macrophages were first infected with NL4-3.HSA.R+E- (Vpr^+^, Env^-^) [43], a strain with the murine heat-stable antigen CD24 (HSA) gene inserted into the *nef *region to facilitate the identification of HIV-1-positive cells by surface CD24 staining. NL4-3.HSA.R+E- was also pseudotyped with the VSV-G envelope to limit viral replication to a single round so that analysis could be performed by limiting cytolysis. The HIV-1-infected cells were further superinfected at 24 hours with the lentivirus vNL-AnlO-RRE-SA. As shown in Fig. 5B, HIV-1-infected macrophages without vNL-AnlO-RRE-SA superinfection generated 12.2% HIV-1-positive cells, while cells superinfected with vNL-AnlO-RRE-SA showed a reduction in HIV-1-positive cells to 3.8%. Additionally, HIV-1-infected macrophages treated with a concentrated dose of vNL-AnlO-RRE-SA generated only 0.27% of HIV-1-positive cells, demonstrating that the AnlO lentivirus has the capacity to diminish the HIV-1-positive cell population. The direct killing of HIV-1-positive macrophages was also consistent with a decrease in the p24 level in the culture supernatant (data not shown).

The selective reduction of HIV-1-positive macrophages did not result from possible non-specific killing of macrophages by the AnlO lentiviral vector. When healthy macrophages were identically treated with the concentrated vNL-AnlO-RRE-SA, or with a control empty vector virus, vNL-RRE-SA, we did not observe differences in cytolysis during a window of two weeks, based on propidium iodide (PI) staining and flow cytometry analysis (Fig. 5C). In contrast, in a control, when puromycin (500 ng/ml) was added into the cell culture to induce non-specific killing, cytolysis was quickly detected within 2 days, and it reached 25% of the cells at day 6 (Fig. 5C).

**Figure 6:**
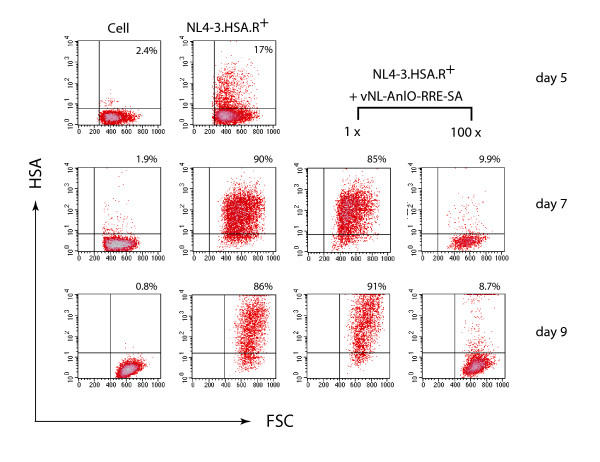
**Selective reduction of HIV-1-infected T cells by the lentiviral vector carrying *anlO***. One million CEM-SS cells were first infected with the replication-competent virus NL4-3.HSA.R+ (250 ng p24 per million). Aliquots of the infected cells were then superinfected 24 hours later with two different doses of vNL-AnlO-RRE-SA (for the 1 × dosage, m.o.i. 0.5 – 1). The extent of spreading HIV infection was measured by mouse CD24 (HSA) staining using a PE-labeled rat monoclonal antibody against HSA. Flow cytometry analyses were performed at day 5, 7, and 9 post HIV infection. Shown are the mouse CD24 staining (HSA) (y-axis) and the Forward Scatter (FSC) (x-axis) of the cells. The second dose of vNL-AnlO-RRE-SA was 100 fold (100 ×) higher than the first one (1 ×). Uninfected cells (Cell) were similarly stained and used as a control.

We further extended our study to test whether the Rev-dependent AnlO lentivirus is capable of killing infected T cells and inhibiting viral spreading. A human CD4 T cell line, CEM-SS, was first infected with a replication-competent virus, NL4-3.HSA.R+ (Vpr^+^, Env^+^) [43]. Following infection for 24 hours, cells were superinfected with two different doses of the Rev-dependent AnlO lentivirus, vNL-AnlO-RRE-SA(VSV-G). Cells were continuously cultured for more than a week, and HIV-1 spread was monitored by surface staining of mouse CD24 expression. As shown in Fig. 6, HIV-1 replication resulted in an infection spreading to 90% of T cells within one week. Superinfection with the low dose of vNL-AnlO-RRE-SA(VSV-G) was not capable of competing with the virus and inhibiting its replication. However, at the high dosage (100-fold), vNL-AnlO-RRE-SA(VSV-G) effectively limited the HIV spread to below 10% of T cells (Fig. 6). These results demonstrate that the Rev-dependent AnlO lentivirus is capable of inhibiting HIV-1 spread by killing of infected cells. Our data also demonstrate that AnlO is an effective toxin both in macrophages and in T cells.

## Discussion

Despite the success of HAART in inhibiting viral replication, the maintenance of HIV-1 in both T cells and macrophages permits viral persistence. In particular, cells from the macrophage lineage, with their ability to resist HIV-1-mediated killing while sustaining a life-long competence for viral production, remain a significant impediment to HIV eradication. Simply waiting for these cells to naturally decay is not an option, given that drug resistance will likely evolve. Novel complementary approaches to specifically target persistently infected cells are urgently needed.

In this report, as a proof of concept, we established a system to specifically target HIV-1-infected macrophages and T cells by utilizing a Rev-dependent lentiviral vector carrying *anl*O. Lentiviruses are unique in their capacity to infect terminally differentiated cells such as macrophages. The Rev-dependency of this vector further limits *anl*O expression to HIV-1-positive cells. The choice of *anlO *as the primary therapeutic gene is based on our demonstration that AnlO exhibits cytolytic activity in macrophages, and that this cytolytic activity can be inhibited by the presence of small quantities of cholesterol such as those present in human plasma. These properties make AnlO an attractive candidate for the safe use of suicidal viral vectors with minimal secondary effects on nontarget cells. We demonstrate that selective killing of HIV-1-infected cells can be achieved with this lentiviral vector while retaining the healthy cell population.

Future application of any therapeutic strategy likely involves simultaneous targeting of multiple viral reservoirs, since macrophages are not the only cells harboring HIV. CD4 T cells are the other major targets of HIV-1. Although productive HIV-1 replication directly kills CD4 T cells, it has been well-documented that some infected T cells can survive virus-mediated killing and revert to a resting memory T cell phenotype [52]. These cells constitute another major reservoir of HIV-1. Many of the previous experimental and clinical attempts have focused on purging this reservoir. A major hurdle to targeting T cells is the lack of viral activity in the absence of cellular stimulation. Chemokines such as IL-2 and IFN-γ have been used in conjunction with HAART to stimulate resting T cells in hopes of "flushing out" this latent virus [26-29]. Decay of the viral reservoir following these treatments would depend on infected cells being killed either by the virus itself or by some immune effector mechanisms. Nevertheless, results from clinical studies so far have not demonstrated significant success in reducing the pool of infected T cells [26,28,29]. The Rev-dependent lentivirus also has its limitations in targeting HIV-1-infected resting T cells, since it is unlikely that a functional amount of Rev exists in resting T cells. Nevertheless, there is a possibility that the Rev-dependent lentivirus could be used in conjunction with chemokine stimulation to target infected T cells.

Although the Rev-dependent AnlO lentiviral vector has demonstrated high specificity in cell culture conditions, future studies in animal models need to address several critical issues, particularly possible bystander effects and non-specific expression of *anlO in vivo*. In cell culture conditions, we have not observed non-specific or bystander killing by the lentiviral vector. In the absence of HIV-1, the Rev-dependent *anlO *vector can be stably maintained in the healthy cell population for extended periods of time (Fig. 4E), an indication of lack of *anlO *gene expression and cytotoxicity in the absence of Rev. However, since there are multiple cell types present in the body, the general effects of *anlO *expression are not known. Additionally, possible mobilization of the AnlO vector in the presence of HIV-1 must also be determined. Although limited mobilization of some lentiviral vectors to non-target cells in the body has been viewed as beneficial [53], mobilization of a vector carrying a toxin would be different.

There is also a possibility that the AnlO lentiviral vector may combine with HIV-1 to generate a replication competent virus, although the rate could be very low [54,55]. In retroviruses, recombination is mediated through template switch during reverse transcription, a process requiring template fidelity [56]. Because of the template variations, non-homologous recombination usually generates large deletions in the viral genome. This is particularly true for the replication-defective oncogenic retroviruses, which acquire cellular oncogenes through non-homologous recombination. However, there are some strains of oncogenic retroviruses, such as those of Rous sarcoma virus, that are replication-competent [57]. Therefore, non-homologous recombination could lead to the generation of a replication-competent HIV-1 with the insertion of *anlO*. Nevertheless, even if this occurred, the *anlO *recombinant virus would not be expected to have a replication advantage over the parental wild-type virus. The cytolytic activity of AnlO would limit the spread of the recombinant virus. This is also consistent with the fact that while retroviruses can acquire many cellular genes, it is rare for them to maintain a cellular pro-apoptotic gene. On the contrary, it is common for the virus to preserve an oncogene that can promote cell survival and provide the virus a replication advantage.

Viral integration-mediated mutagenesis is another issue that requires future attention, should large quantities of viral particles be injected. Currently, we are also examining an unintegrating Rev-dependent lentiviral vector for targeting HIV-1-infected cells. We previously demonstrated that the unintegrated HIV-1 DNA can mediate transient gene expression in T cells [58] and persistent transcription in human macrophages [59]. Unintegrating lentiviral vectors have been recently used to transduce non-dividing cells, such as ocular or neuronal cells, for gene therapy [60-62]. These vectors have demonstrated a surprising efficiency in mediating gene expression from internally promoted transgenes. Such vectors would be advantageous for minimizing integrationmediated mutagenesis. Future practical application of the AnlO lentiviral vector likely involves multiple injections in combination with anti-retroviral drugs to halt HIV spread. A more effective approach to specifically target tissues harboring large quantities of infected cells may be achieved with localized injection. Yet another method to enhance target specificity would be to engineer the envelope of the vector to carry gp120 binding domains so that it can only bind and enter HIV-1-positive cells. Such a possibility has been demonstrated previously in the laboratory [63].

Even with many of the limitations described above, a lentiviral vector has recently been tested for *ex vivo *delivery of an antisense gene against the HIV envelope into CD4 T cells [64]. While a phase I clinical trial has provided encouraging data for its safe and feasible application in the treatment of HIV-1-infected patients [64], a drawback of the infusion of gene-modified CD4 T cells is the lack of access to the macrophage reservoir. Persistence of viral replication in the body is expected even with the adoptive transfer of HIV-1-resistant T cells. Injection of lentiviral particles may have the benefit of targeting multiple viral reservoirs. Furthermore, *in vivo *delivery of conditionally replicating lentiviral vector may have additional immunological benefits as an attenuated vaccine. Our data clearly demonstrate that Rev-regulated gene expression can be utilized as a vehicle for the selective delivery of novel therapeutic genes.

## Methods

### Cloning of the *anlO *gene from *B. anthracis*

pNL-GFP-RRE-SA has been previously described [34,36]. pNL-AnlO-GFP-RRE-SA was constructed by inserting the B*amHI*-X*hoI *fragment of pAnlO, a plasmid containing the *anlO *gene of the 34F2 (Sterne) strain of *B*. *anthracis *(kindly provided by Dr. Serguei Popov), into the B*amHI*-S*alI *sites of pNL-GFP-RRE-SA. pNL-AnlO-RRE-SA was constructed by further deletion of the GFP ORF with restriction digestion. Successful cloning of the *anlO *gene was further confirmed by DNA sequence analysis. The packaging construct, pCMVΔ8.2, was kindly provided by Dr. Dider Trono. pCAGGSSF162gp160 [51] was obtained from the NIH AIDS Research & Reference Reagent Program, NIAID, NIH.

### Virus production

The HIV-1 strains, NL4-3.HSA.R+E-(VSV-G) and the replication-competent NL4- 3.HSA.R+E+ [43] ("R" represents the Vpr gene and "E" represents the viral envelope gene) were provided by the NIH AIDS Research & Reference Reagent Program, NIAID, NIH. In both viruses, the murine heat-stable antigen CD24 (HSA) gene was inserted into the *nef *region that allows HIV-1-positive cells to be monitored by surface staining of HSA. Viruses were produced by transfection of HEK293T cells (provided by the NIH AIDS Research & Reference Reagent Program, NIAID, NIH), using Lipofectamine™ 2000 (Invitrogen, Carlsbad, CA) as recommended by the manufacturer. HIV-1 titer was determined using an indicator cell line, Rev-CEM, as previously described [36]. The Rev-dependent GFP and AnlO lentiviruses, vNL-GFP-RRE-SA and vNL-AnlO-RRE-SA, were produced by cotransfection of HEK293T cells with calcium phosphate (Promega, Madison, WI). Briefly, two million cells were cultured in a petri dish and cotransfected with 10 μg of either pNL-GFP-RRE-SA or pNL-AnlO-RRE-SA, 7.5 μg of pCMVΔ8.2, and 2.5 μg of the envelope constructs. Transfected cells were cultured overnight, and then the supernatant was removed and replaced with 10 ml fresh DMEM plus 10% heat-inactivated fetal bovine serum (FBS). For the production of vNL-AnlO-GFP-RRE-SA, 10 nM 6-boc orthinine amide-β-cyclodextrin (kindly provided by Dr. Vladimir Karginov) was also added into the medium to prevent cell lysis by *anlO *expression. Viruses were harvested at 48 hours and then concentrated by multiple rounds of concentration through anion exchange columns and size-exclusion columns. Concentrated virus was divided into 50 μl aliquots and stored at -80°C. Viral p24 level was determined using p24 ELISA assay (Beckman Coulter, Miami, FL). The p24 levels of concentrated viruses were between 2 and 10 μg/ml. The titer of vNL-GFP-RRE-SA was measured directly on an HIV-1-positive cell line, J1.1 [50] (provided by the NIH AIDS Research & Reference Reagent Program, NIAID, NIH), which was cultured in 50 ng/ml PMA (phorbol myristate acetate) to stimulate HIV-1 activity. GFP-positive J1.1 cells were enumerated on FACSCalibur (BD Biosciences, San Jose, CA). The titer of vNL-AnlO-GFP-RRE-SA cannot be measured directly due to its cytolytic activity, and thus was estimated based on p24 levels, using the titer of vNL-GFP-RRE-SA as a reference.

### Cells and viral infection

CEM-SS was acquired from the NIH AIDS Research & Reference Reagent Program, NIAID, NIH. Macrophages were differentiated from human monocytes from the peripheral blood of HIV-1 negative donors. All protocols involving human subjects were reviewed and approved by the George Mason University IRB. Briefly, two million peripheral blood mononuclear cells were plated into each well of six plates in serum-free RPMI medium for one hour. Adherent cells were cultured in RPMI plus 10% FBS and 10 ng/ml macrophage colony-stimulating factor (M-CSF) (R&D System, Minneapolis, MN) for two weeks with medium change every two days. Differentiated macrophages were infected with NL4-3.HSA.R+E-(VSV-G) at a multiplicity of infection of 0.1. Viral replication was monitored by cell surface staining of mouse CD24 antigen and p24 ELISA (Beckman Coulter, Miami, FL). CEM-SS T cells were infected with replication competent HIV-1 NL4-3.HSA.R+E+. Aliquots of infected cells were superinfected at 24 hours with vNL-AnlO-RRE-SA using different doses of concentrated virus. HIV-1-positive cell were monitored by immunostaining and flow cytometry on a FACSCalibur (BD Biosciences, San Jose, CA).

### Immunofluorescent staining

One half to one million infected cells were removed from the culture dish and washed once with cold PBS, centrifuged for 5 minutes at 400 × g and resuspended in 400 μl cold staining buffer (PBS plus 1% BSA). Nonspecific binding was blocked by adding 5 μl Rat IgG (10 mg/ml) (Jackson Laboratories Inc., Westgrove, PA). HIV-1-positive cells were stained with 2 μl of PE-labeled Rat Anti-Mouse CD24 (Southern Biotech, San Diego, CA). For isotype control staining, PE-labeled Rat IgG_2a _(BD Biosciences, San Jose, CA) was used. Stained cells were incubated on ice for 30 minutes and then washed with cold PBS plus 1% BSA and resuspended in 500 μl of 1% paraformaldehyde for flow cytometry analysis on a FACSCalibur (BD Biosciences, San Jose, CA).

### PCR amplification

Total cellular DNA was purified using a Wizard Genomic DNA purification kit as recommended by the manufacturer (Promega, Madison, WI). For the detection of the AnlO lentiviral vector in infected cells by PCR, the forward primer 5'GGTTAGACCAGATCTGAGCCTG 3' and the reverse primer 5'GTGTTTCTGCCATGGTAAGG 3' were used. PCR was carried out in 1 × Ambion PCR buffer, 125 μM dNTP, 50 pmol each primer, 1 U SuperTaq Plus (Ambion Inc. Austin, TX) with 35 cycles at 94°C for 10 seconds, 68°C for 50 seconds. For relative quantification of the PCR reaction, the cellular β-actin pseudogene was also amplified with primers from the QuantumRNA β-actin Internal Standards, using conditions as suggested by the manufacturer (Ambion Inc. Austin, TX). Briefly, the PCR was carried out in 1 × Ambion PCR buffer, 125 μM dNTP, 1 U SuperTaq Plus (Ambion Inc. Austin, TX) with 25 cycles at 94°C for 20 seconds, 68°C for 60 seconds.

## Competing interests

The authors declare that they have no competing interests.

## Authors' contributions

JY carried out the T cell killing studies, participated in the specificity test in macrophages and the manuscript preparation. ZT performed the cloning, lentiviral vector production and the macrophage killing studies. QY participated in the cloning and sequence analysis. DY carried out the PCR amplification, participated in the specificity test in macrophages. YW conceived of the study, participated in its design and coordination, and the specificity studies on the Rev-dependent vector, and drafted the manuscript. All authors read and approved the final manuscript.
